# Improved Tissue Culture Conditions for Engineered Skeletal Muscle Sheets

**DOI:** 10.1155/2013/370151

**Published:** 2013-02-28

**Authors:** Sara Hinds, Natalia Tyhovych, Clint Sistrunk, Louis Terracio

**Affiliations:** Department of Basic Sciences, New York University, 345 East 24th Street, New York, NY 10010, USA

## Abstract

The potential clinical utility of engineered muscle is currently restricted by limited *in vitro* capacity of expanded muscle precursor cells to fuse and form mature myofibers. The purpose of this study was to use isotropic skeletal muscle sheets to explore the impact of (1) fibroblast coculture and (2) fibroblast-conditioned media (fCM) on *in vitro* myogenesis. Muscle sheets were prepared by seeding varying ratios of skeletal myoblasts and fibroblasts on a biomimetic substrate and culturing the resulting tissue in either control media or fCM. Muscle sheets were prepared from two cell subpopulations, (1) C2C12 and NOR-10 and (2) primary neonatal rat skeletal muscle cells (nSKM). In C2C12/Nor-10 muscle sheets fCM conferred a myogenic advantage early in culture; at D1 a statistically significant 3.12 ± 0.8-fold increase in myofiber density was observed with fCM. A high purity satellite cell population was collected from an initially mixed population of nSKMs via cell sorting for positive **α**7-integrin expression. On D6, tissue sheets with low fibroblast concentrations (0 & 10%) cultured in fCM had increased average myofiber density (4.8 ± 0.2 myofibers/field) compared to tissue sheets with high fibroblast concentrations (50%) cultured in control media (1.0 ± 0.1 myofibers/field). Additionally, fCM promoted longer, thicker myofibers with a mature phenotype.

## 1. Introduction

Pathological abnormalities of the facial musculature can occur as a consequence of congenital defects, traumatic injuries, or surgical ablations [1–4]. Skeletal muscle possesses an innate capacity to self-regenerate following minor injury by recruiting quiescent muscle precursor cells to the site of injury where they then fuse and form mature myofibers [5]. Of the resident stem cell populations found in native muscle, satellite cells are believed to be the principle progenitors during the repair of small tissue defects [6, 7]. Unfortunately, the ability of satellite cells to repair large defects is minimal and exogenous reconstruction may be necessary to restore functionality. Skeletal muscle tissue engineering is a promising therapeutic option for large-scale skeletal muscle myopathies. The ultimate goal of the tissue engineering approach is to develop skeletal muscle with appropriate tissue morphology and functionality that can be implanted *in vivo* to enhance the architecture and contractility of defective muscle [1, 8–10]. 

Satellite cells have been proposed as a favorable autologous stem cell source for tissue-engineered constructs because of their role in myoblast differentiation [11]. Mononuclear satellite cells, which can be found on the external surface of native muscle fibers, remain predominantly in a quiescent state until the cells become mitotically active in response to transcription factors released during periods of muscle fiber injury [1–4]. *α*7-Integrin has been shown to be a primary cell marker that signals the linearization of replicating satellite cells and is known for upregulating myoblast fusion in adult muscle cells in the event of injury [12]. Isolation techniques based on *α*7-integrin immunotagging can be used to collect cells from a muscle biopsy. Utilizing fluorescence-activated cell sorting (FACS), a sample of mixed skeletal muscle can be sorted by the cells expressing *α*7 for myoblast enrichment [12, 13]. Current techniques for the expansion and differentiation of satellite cells *in vitro *have been hindered by the expanded cells' lack of ability to fuse into myotubes and express normal force generation. The pretransplant muscle tissues have underperformed in excitability and contractility testing [14]. The scope of this study was to develop a tissue culture that will improve the myogenic process for bioengineered isotropic skeletal muscle sheets based on the role of fibroblasts and the effect of paracrine factors they release for myotube formation. 

The physiological relevance of muscle fibroblasts is associated with the regulation of myogenesis. The normal function of fibroblasts is to synthesize the ECM, specifically collagen fibers and laminin for mechanical function [7, 15]. The presence of laminin is important for structural integrity and various cellular responses. Laminin transduces signals within the ECM for secondary myofiber formation by causing allosteric changes to *α*7. The integrin guides motile primary myoblasts to laminin-rich sites along the basement membrane promoting isotropic orientation [13, 16]. In response to tissue damage, fibroblasts become more transcriptionally active for the release of cytokines and growth factors, a highly ordered regenerative process [17]. After injury, satellite cells and fibroblasts develop in close proximity, each regulating the other. Fibroblasts adjust the expansion of satellite cells by repressing their terminal differentiation into primary myoblasts. During early myogenesis, satellite cells reciprocate fibroblast regulation for connective tissue development. However, satellite cells begin to monitor fibroblast expansion through a negative feedback loop in late myogenesis to prevent excess fibrosis. Excessive ECM can impede structural integrity and regeneration of new tissue [15]. The cellular response to skeletal muscle injury also includes an increase in intracellular paracrine signaling within the tissue. The paracrine proteins expressed are a variety of fibroblast growth factors (FGFs) and insulin-like growth factors (IGFs), predominantly FGF-1, FGF-2, and IGF-1. FGF-1 and -2 are functionally relevant to the repression of terminal differentiation in primary myoblasts [18]. The signaling of IGF-1 has been shown to activate numerous biochemical pathways associated with skeletal muscle hypertrophy induction [19, 20]. 

## 2. Materials and Methods

### 2.1. Isolation of Primary Satellite Cells and Cell Culture

Primary rat neonatal satellite cells were isolated as previously described [8] using a modification of the Bischoff's protocol [21, 22]. Briefly, muscle tissue from the hind limbs of 2-3-day-old rats was excised, minced, separated from connective tissue, incubated with 1.25% protease solution (9.5 g Krebs buffer, 10 mL of 2 M HEPES, 0.5 mL phenol red, 1.25 mg/mL protease, in 1 L filtered H_2_O gassed for 20 mins with CO_2_) for 90 min while rocking, twice centrifuged, resuspended in SK culture medium (DMEM, 25% fetal bovine serum, 1% antibiotics) with 600 U DNase enzyme, and then preplated on 150 mm culture dish for 120 min. The unattached cells were harvested and cultured in SK medium for 2 days and expanded in growth medium (DMEM, 20% fetal bovine serum, 1% antibiotics) before cell sorting.

As a secondary cell source, Murine C2C12 myoblasts (ATCC), a cell line that originated from normal adult C3H mouse leg muscle, were cultured in growth medium for less than 4 passages. Murine Nor-10 skeletal muscle fibroblasts (ATCC), a cell line that originated from normal C57BL/10 mouse muscle, were cultured in growth medium. 

### 2.2. Satellite Cell Purification Utilizing FACS

 Cultures of cells isolated from rat skeletal muscle were sorted using fluorescence-activated cell sorting. Selection was based on the expression of an extracellular epitope of the muscle-specific *α*7 integrin protein (9 MBL International).

### 2.3. Preparation of Fibroblast-Conditioned Medium

 Nor-10 and alpha-7 negative muscle fibroblasts were cultured separately in growth medium until ~80% confluence was achieved and then switched to differentiation medium (DMEM, 10% horse serum, 1% antibiotics) for 3 days. The conditioned medium (CM) was collected, filtered, and stored at −4° until needed for tissue culture at which point it was thawed, sterilized, and combined 1 : 1 with fresh differentiation medium. Nor-10-CM and alpha-7-negative-CM were exclusively used on tissue sheets composed of C2C12 myoblasts and alpha-7 positive satellite cells, respectively. 

### 2.4. Assembly of Muscle Tissue Sheets

 Tissue culture dishes (12 well) were treated with (1 mg/mL) laminin for 1 hour prior to cell seeding. Isotropic muscle sheets were prepared by simultaneously seeding myoblasts and fibroblasts (total of 23 × 10^3^ cells/mL) in the following combinations: (1) 100%  C2C12, (2) 90% C2C12 and 10% Nor-10, (3) 50% C2C12 and 50% Nor-10, (4) 100%  *α*7+ satellite cells, (5) 90%  *α*7+ satellite cells and 10%  *α*7-muscle fibroblasts, and (6) 50%  *α*7+ satellite cells and 50%  *α*7-muscle fibroblasts. The tissue sheets were cultured in growth medium for 3 days and then switched to either fibroblast-conditioned medium or control medium (DMEM, 10% Horse serum, 1% antibiotics). C2C12/Nor-10 tissue sheets were cultured for 1 day in differentiation medium and then analyzed for early culture myogenesis while *α*7+/*α*7− tissue sheets were cultured for 6 days in differentiation medium and then analyzed for late culture myogenesis. 

### 2.5. Quantification of Myofiber Density

 Live culture images (4 fields/well) were captured with a phase contract microscope (Nikon Instruments). Total myofibers/images were counted and averaged for each well at time points D1, for C2C12/Nor-10 tissue sheets, and D6, for *α*7+/*α*7− tissue sheets. 

### 2.6. Immunostaining

 Muscle tissue sheets were fixed with 4% formaldehyde at room temperature, permeabilized with 0.1% Triton X, blocked with BSA, and incubated with Desmin primary antibodies (BD Biosciences) for 45 mins and incubated in secondary antibodies (BD Biosciences) together with a Hoechst nuclear dye (Invitrogen) for 1 hr. Images were acquired using a fluorescence microscope (Nikon Instruments). 

### 2.7. Statistics

 Data are expressed as mean ± SE. Statistical significance was determined by two-way ANOVA with post hoc Tukey's test. Differences were considered to be significant when *P* < 0.05. 

## 3. Results and Discussion

It is well established that fibroblasts play a critical role in skeletal muscle formation and function, while also being instrumental in myopathogenesis [7, 15, 17]. Our methods were used to analyze the effect of fibroblasts on myogenesis of tissue skeletal muscle sheets both directly, with addition of fibroblast to tissue culture, and indirectly, with the application of fibroblast-conditioned media (fCM). Our aim for this study was to harness the myogenic capabilities of satellite cells *in vitro *by providing them with a biomimetic environment complete with fibroblast paracrine factors that they are likely to receive *in vivo*. 

**Figure 1:**
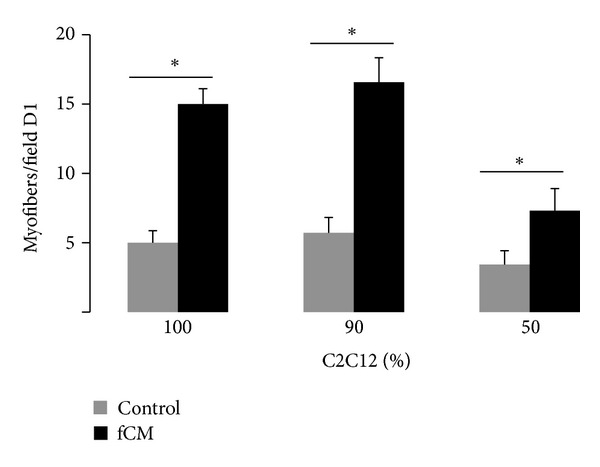
Myofiber density in C2C12/Nor-10 tissue sheets with varied initial cell concentration; 100% C2C12 with 0% Nor-10, to 90% C2C12 with 10% Nor-10, to from 50% C2C12 with 50% Nor-10. Bars show *n* = 8 tissue sheets per group. “∗” statistically significant between the indicated pairings, *P* < 0.05.

Muscle tissue sheets were successfully prepared from a coculture of C2C12 myoblasts and Nor-10 fibroblasts and analyzed at D1 for early culture myogenesis. All tissue sheets cultured in control medium had comparable myofiber density, an average of 5.0 ± 0.58 myofibers/field (Figure 1). Alternatively, the tissue sheets cultured in fCM were largely impacted by the concentration of Nor-10 fibroblasts. In fCM, the highest myofiber densities, 15.1 ± 1.1 & 16.9 ± 1.8 myofibers/field, were observed in tissue sheets with the lowest concentration of Nor-10 cells, 0% & 10%, respectively, while the myofiber density of tissue sheets with 50% Nor-10 was significantly reduced, 7.8 ± 1.6 myofibers/field (Figure 1). Notably, for each coculture ratio a statistically significant increase in myofiber formation was seen in tissue sheets cultured in fibroblast-conditioned medium (fCM). As compared to control medium, fCM conferred a 4.2 ± 1.5-, 3.3 ± 0.5-, & 4.8 ± 2.1-fold increases in myofiber density were seen in the tissue sheets with 100%, 90%, & 50% C2C12 cells, respectively (Figure 1). 

**Figure 2:**
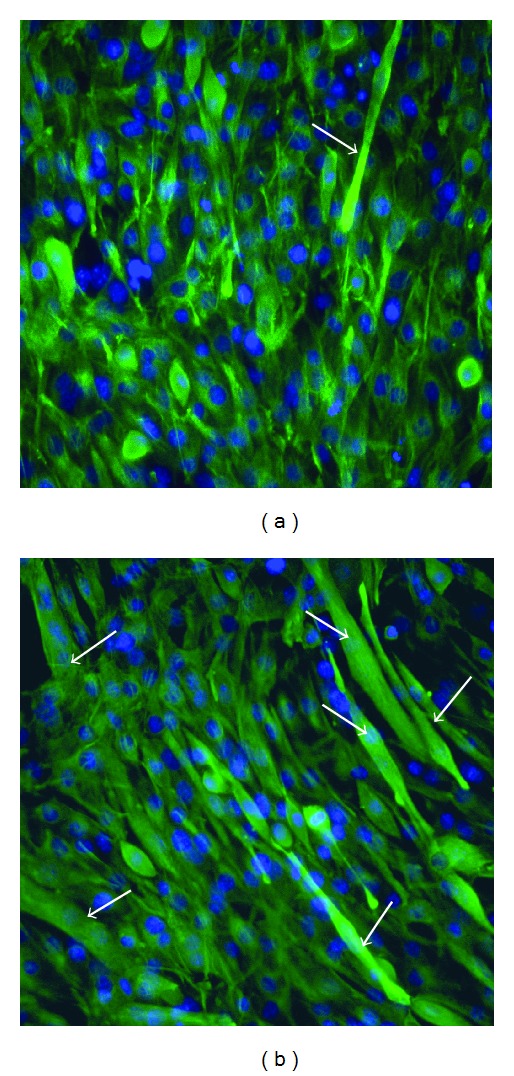
Multinucleated myofibers in representative muscle tissue sheets (90% C2C12 and 10% Nor-10) cultured in (a) control medium or (b) fCM. Arrows indicate myofibers that stained positive for muscle-specific Desmin (green) and nuclei (blue). Both tissue sheets were fixed at D1 of differentiation.

 Immunoflourescence staining of representative images from the 90% C2C12 coculture condition (Figure 2) further reveled that tissue sheets cultured in fCM had improved myofiber formation as well as enhanced desmin expression, a marker of early myogenesis [23]. Additionally, multinucleated myofibers were more abundant and had a larger fiber diameter in the tissue sheets cultured in fCM (Figure 2(b)) as compared to control medium indicative of a more mature and functional phenotype. 

**Figure 3:**
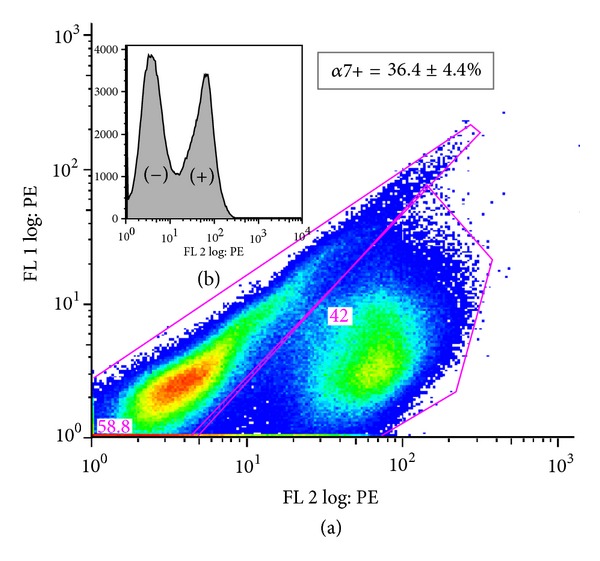
Expression of *α*7 in a mixed cell population. (a) *α*7 PE (b) expression histogram.

As a step toward translation, the effect of fCM was explored further with primary isolated neonatal rat skeletal muscle cells (nSKM). Our aim was to selectively sort muscle satellite cells, which are widely considered to be a more clinically relevant cell source as compared to immortalized cell lines [11]. The initial nSKM isolation yielded a mixed population of cells with varying phenotypes, some spindle shaped and some flattened with many pseudopod extensions. Fluorescence-activated cell sorting (FACS) resulted in two populations of cells, (1) *α*7 positive cells (*α*7+) and (2) *α*7 negative cells (*α*7−) (Figure 3). Satellite cells, known to be *α*7+, accounted for 36.4 ± 4% of the initial cell population [12, 13]. After sorting, the collected *α*7+ cells had a purity of 91%. The *α*7− cells were passaged several times to ensure high fibroblast purity. Ultimately, autologous muscle biopsies will be a desirable source for satellite cells and muscle fibroblasts to be used in tissue-engineered constructs for therapeutic muscle augmentation, and these methods demonstrate a step toward clinical application. 

**Figure 4:**
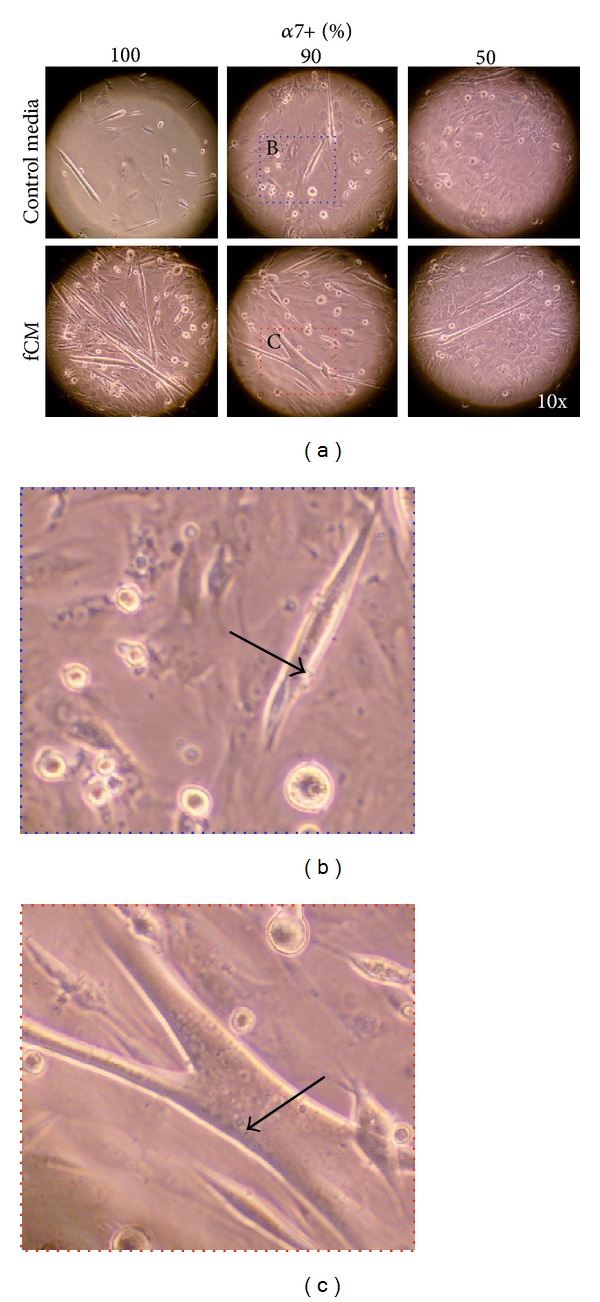
Morphology of muscle tissue sheets. (a) Live culture images from tissue sheets with varying *α*7+ concentration (100, 50, 90%) in either control media (top panel) or fCM (middle panel). Enlarged representative sections from 90%  *α*7+ tissue sheets cultured in (b) control media and (c) fCM demonstrate that the myofibers in fCM are longer and thicker.

**Figure 5:**
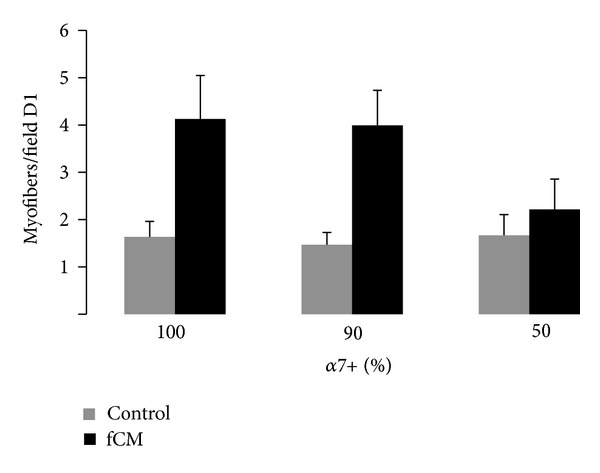
Myofiber density in *α*7+/*α*7− tissue sheets with varied initial cell concentration; 100%  *α*7+ with 0%  *α*7−, 90%  *α*7+ with 10%  *α*7−, to from 50%  *α*7+ with 50%  *α*7−. Bars show *n* = 3 tissue sheets per group. “∗” statistically significant between the indicated pairings, *P* < 0.05.

 Muscle tissue sheets were successfully prepared from a coculture of primary satellite cell (*α*7+) and muscle fibroblasts (*α*7−) (Figure 4). In culture, tissue sheets with fCM were more active and showed spontaneous contractility, indicating mature and well-assembled contractile machinery, while those cultured in control media did not have spontaneous contractile activity. The resulting tissues were then analyzed at D6 for late culture myogenesis. Similar to C2C12 muscle sheets, the primary cell muscle sheets cultured in control media had minimal dependence on fibroblast concentration with an average of 1.6 ± 0.2 myofibers/field (Figure 5). Alternatively, the sheets cultured in fCM had a greater dependence on fibroblast concentration and those muscle sheets with the lowest concentration of fibroblasts, 100% & 90%  *α*7+, tended to have the greatest myofiber density, 4.1 ± 0.9 & 4.0 ± 0.7 myofibers/field, respectively, compared to 50%  *α*7+ sheets, 2.2 ± 0.6 myofibers/field (Figure 5). Again, for each coculture ratio a significant increase in myofiber formation was seen in tissue sheets cultured in fCM as compared to control medium. 2.5 ± 0.3-, 2.7 ± 0.3-, and 1.4 ± 0.3-fold increases in myofiber density were seen in the tissue sheets with 100%, 90% and 50%  *α*7+ cells, respectively (Figure 5). Finally, the muscle sheets cultured in fCM were composed of longer myofibers with distinctly larger diameter (Figure 4(c)).

Direct addition of fibroblast in culture had a less significant impact on myofiber density compared to conditioned media. The impact of initial fibroblast concentration was amplified in the presence of fCM. This amplification is likely the result of increased cell proliferation induced by the growth factors found in conditioned media. High fibroblast proliferation could lead to a condition that mimics fibrosis and would inhibit myofiber formation. 

Interestingly, the primary satellite cells had a lower overall capacity to form myofibers as compared to C2C12 cells, which readily assemble confluent sheets by D2. Satellite cells may be more dependent on exogenous stimulus to form complete muscle sheets and therefore fCM may represent an important addition to a successful muscle bioreactor. Fibroblasts can be easily isolated from tissue biopsy such that fibroblast-conditioned media would be available along with an autologous cell isolate. 

While the present study explores the effect of fibroblast-derived paracrine factors on myogenesis, it is also known that proinflammatory cytokines secreted by monocytes and macrophages may play an important role during *in vivo* satellite cell recruitment and differentiation [24]. In a muscle tissue engineering setting the role of proinflammatory mediators is less clear. It may be desirable to further examine the effect proinflammatory cytokines on tissue-engineered skeletal muscle.

The results of this study demonstrate the effectiveness of fCM in promoting myofiber formation. In the future we will apply this new tissue culture condition to 3D-engineered muscle construct with biomimetic tissue organization, for which we have previously developed a methodology [8], in an attempt to improve the muscle tissue formation and functionality. 

## 4. Conclusions

Engineered skeletal muscle sheets were successfully prepared from a two distinct subpopulations of myoblasts. Initial trials of early tissue culture with myoblast and muscle fibroblast cell lines showed a statistically significant increase in myofiber density when the engineered tissue was cultured in fibroblast-conditioned media (fCM). As a step toward translation, we then explored the impact of fCM on freshly isolated satellite cells. At D6, fCM treatment had a qualitative and quantitative impact on myofiber formation for tissue sheets prepared with primary nSKMs. From these results, we conclude that conditioned media can be used to potentiate myogenesis *in vitro *through improved differentiation of skeletal muscle precursor cells. Paracrine factors released by fibroblasts may represent an important target for potentiating myogenesis in engineered skeletal muscle constructs.
